# Identification of DNA primase inhibitors via a combined fragment-based and virtual screening

**DOI:** 10.1038/srep36322

**Published:** 2016-11-02

**Authors:** Stefan Ilic, Sabine R. Akabayov, Haribabu Arthanari, Gerhard Wagner, Charles C. Richardson, Barak Akabayov

**Affiliations:** 1 Department of Chemistry, Ben-Gurion University of the Negev, Beer-Sheva, 8410501, Israel; 2 Department of Biological Chemistry and Molecular Pharmacology, Harvard Medical School, 240 Longwood Ave., Boston, Massachusetts 02115, USA

## Abstract

The structural differences between bacterial and human primases render the former an excellent target for drug design. Here we describe a technique for selecting small molecule inhibitors of the activity of T7 DNA primase, an ideal model for bacterial primases due to their common structural and functional features. Using NMR screening, fragment molecules that bind T7 primase were identified and then exploited in virtual filtration to select larger molecules from the ZINC database. The molecules were docked to the primase active site using the available primase crystal structure and ranked based on their predicted binding energies to identify the best candidates for functional and structural investigations. Biochemical assays revealed that some of the molecules inhibit T7 primase-dependent DNA replication. The binding mechanism was delineated via NMR spectroscopy. Our approach, which combines fragment based and virtual screening, is rapid and cost effective and can be applied to other targets.

The complex process of identifying antibacterial compounds begins with the selection of potential targets, which must be essential, selective over human homologues, susceptible to drugs, and with a low propensity to develop rapid resistance^1^. Although bacteria possess approximately 200 essential gene products, only a limited number of these have been exploited as drug targets^2^. DNA replication, which qualifies as a novel drug target, is performed by the replisome, a multi enzyme complex that synthesizes DNA continuously on its leading strand and discontinuously on its lagging strand^3^,^4^. DNA primase, an essential component of the DNA replication machinery of every living cell^5^, synthesizes short RNA primers that are used by DNA polymerase to form the “Okazaki fragments” on the lagging DNA strand. The inhibition of primase, therefore, will halt DNA replication and, as a result, cell proliferation.

Prokaryotic primases (among which is the primase domain of the multifunctional gene 4 protein of bacteriophage T7, the model used in our study) share a conserved primary sequence (Fig. 1a) and are structurally highly similar (Fig. 1b)^5^. In contrast, the profound differences between human and bacterial DNA primases (Fig. 1c) render the latter a selective target for drug design. Specifically, human primase has four subunits^6^ (Fig. 1c, right), while bacterial DnaG usually functions together with the hexameric ring of DnaB helicase (Fig. 1c, left). In addition, sequence homology between the mammalian and bacterial primases is very low^5^. Finally, DnaG possesses an active site for binding nucleotides and a DNA binding module, indicating that bacterial primase is a target for drugs. Despite its high therapeutic potential, however, no clinical candidate inhibitors of DnaG primase have emerged to date.

Historically, the screening process for potential ligands has relied heavily on high throughput screening (HTS). The low effectiveness of HTS in identifying new antibacterial agents^7^, however, led to the emergence of fragment-based screening as a viable alternative route for hit discovery in infectious disease research. Screening of small molecules, whether by fragment screening or HTS, can target key biochemical process or binding to an essential cellular component. Fragment-based screening monitors the binding of smaller molecules from fragment libraries^8^, where the small sizes of the molecules constituting a typical fragment library increases the chances of binding but the strength of that interaction is weak^9^,^10^. Another potential disadvantage for fragment-based screening is the low selectivity of the resulting hits^11^. Although such low affinity-low selective-low weight hits were not believed to indicate the presence of a potentially viable clinical candidate, molecules found by using fragment-based screening are emerging in the late stages of clinical trials^8^. To detect the weak binding affinities (K_D_ ~ μM - mM) on which fragment-based screening relies, saturation transfer difference (STD) spectroscopy is used^12^.

The weak catalytic activity of DNA primase renders the adaptation of a functional assay to HTS a formidable challenge. Here we propose a novel, hybrid method for developing small molecule inhibitors for T7 primase to circumvent the drawbacks of HTS (Fig. 2). Based on the ‘rational design’ philosophy of lead development, our method exploits NMR to identify binders from libraries of fragment molecules. We then use computational methods to construct larger molecules with improved binding/inhibition properties. We show that the use of fragment based virtual screening (FBVS, Fig. 2) can yield potent inhibitors, reduce costs, and provide more advanced information about lead binding properties prior to the medicinal chemistry phase of drug optimization.

## Results

**Figure 1:**
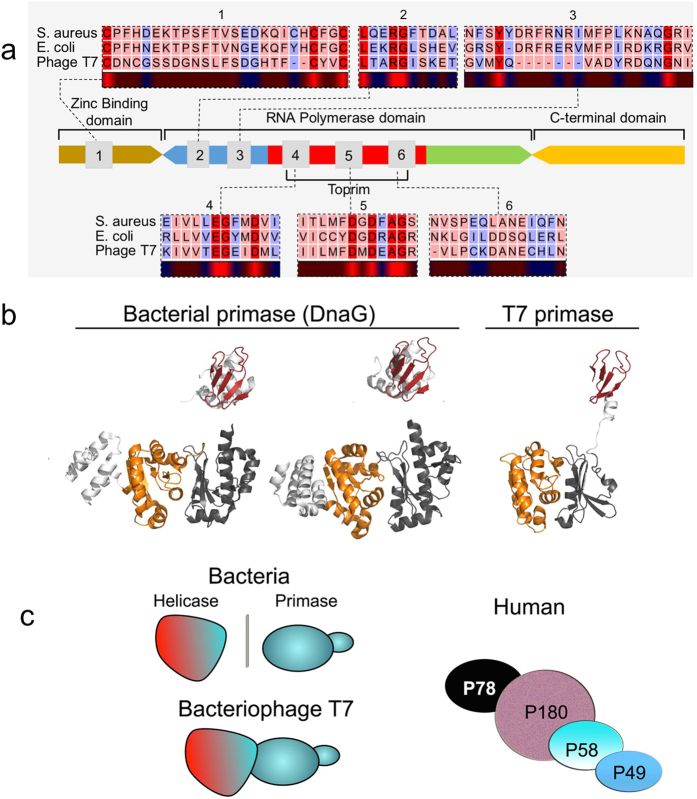
arrangement of DNA primase. (**a**) Domain organization and arrangement of motif sequences of prokaryotic DNA primases (modified from^5^). (**b**) Structural homology of prokaryotic primases. The bacterial DnaG of *Aquifex aeolicus* (PDBID 2AU3^32^, left) and *S. aureus* (PDBID 4E2K^33^, middle) shows structural similarity with the T7 DNA primase, part of the fused helicase-primase gp4 of bacteriophage T7 (PDBID: 1NUI^17^, right). The zinc-binding domain (ZBD) is colored red and the RNA polymerase domain (RPD) is colored yellow-orange. (**c**) Schematic models of prokaryotic primase vs. eukaryotic primase. Left: The structure of bacterial DnaG is similar to that of T7 DNA primase, part of the fused helicase-primase gp4 of bacteriophage T7. A detailed description on the similarity between T7 DNA primase and dnaG bacterial primase is presented in ref. 17. Right: The DNA polymerase α–primase complex from humans consists of four subunits. The p180 subunit is polymerase α, p58 and p49 comprise the primase, and p78 is the fourth, tightly bound subunit.

Bacterial DnaG primase synthesizes RNA primers that are used by DNA polymerase in lagging strand synthesis during DNA replication. Owing to the stark differences between the human and bacterial primases, DnaG primase has long been a target for drug discovery. Here we describe the development of small molecule inhibitors of DnaG using the structurally similar primase domain of the bacteriophage T7 gene 4 protein (Fig. 1b) as a model for the bacterial primase.

To find compounds that do not bind at high affinity but that have potential to become successful leads, we developed a platform for lead discovery comprising several complementary steps: (1) fragment based screening by STD spectroscopy of a small molecule library (Ro3), (2) hit optimization by virtual screening and the generation of a new set of drug-like compounds (Ro5), each of which contains a small molecule found in step 1, (3) docking of the drug-like compounds to the active site of T7 primase and selection of the compounds that will undergo functional and structural assays with the target T7 primase, and (4) further development of lead compounds.

### Fragment Based Virtual Screening (FBVS) – A combined screening approach

**Figure 3:**
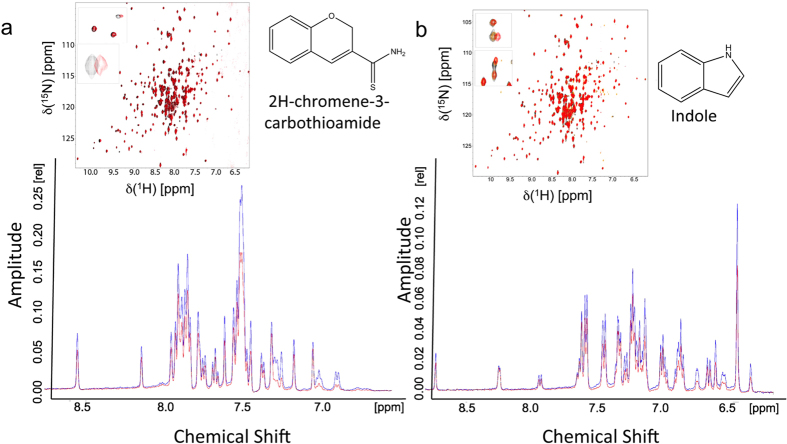
NMR binding assays for fragment molecules. The STD signal is based on the difference, STDDiff, between two recorded spectra: (1) STDon, where the protein target is excited selectively (without exciting the ligand) and the excitation is transferred to the ligand (if it is bound) via spin diffusion of the proton network, and (2) STDoff, i.e., the standard 1D ^1^H NMR spectrum of the fragment molecule (used as a control)^34^. (**a**,**b**) Top: 1D-NMR STD spectra of samples containing 10 scaffold compounds (including 2H-chromene-3-carbothioamide and indole, respectively) and T7 DNA primase. A decrease in the peak intensity after saturation indicates binding of the fragments to the primase. The change at a specific chemical shift value (x-axis) enables the identification of the molecule. insets: 2D ^1^H,^15^N HSQC spectra of ^15^N labeled T7 DNA primase in the presence of the fragments 2H-chromene-3-carbothioamide and indole found by STD spectroscopy. Chemical structure of 2H-chromene-3-carbothioamide and indole are presented.

Growing a fragment molecule into a larger molecule that possesses drug like properties is the bottleneck in fragment-based drug design approaches^13^,^14^. Using the Maybridge Ro3 fragment library containing 1000 fragments, we prepared 100 NMR samples, each containing a mixture of 10 fragments and 50 μM T7 primase. We used 10 fragments per each NMR sample to significantly minimize NMR time. The 10 fragments were chosen to show minimal overlap of their ^1^H chemical shift to allow easy identification of the shifts. The 1D saturation transfer difference spectra of these samples were measured, and fragments that exhibited saturation transfer (evident by a decrease in the peak intensity) were identified. Hits were ranked based on the number of peaks in the ^1^H-NMR spectra with decreased intensities. The small molecule fragments indole and 2H-chromene-3-carbothioamide (Fig. 3) exhibited the strongest binding that, correspondingly, was reflected in the largest decreases in NMR peak intensities (considering the total change in intensity and also the number of affected peaks). Hits were validated by measuring the [^15^N,^1^H] HSQC spectra of ^15^N,D-T7 primase and then evaluating the chemical shift perturbations of the backbone amide resonances upon the addition of the small fragment molecules (Fig. 3). This validation also ensures that the change in peak intensities in the 1D spectra was not a result of additive effects of several fragments.

### Lead optimization and candidate selection

Hits from fragment-based screening are commonly optimized by creating larger compounds with better binding properties, a time and resource intensive step that usually involves medicinal chemistry.

To eliminate the need for a medicinal chemistry phase during the early steps of lead optimization, we used virtual screening where the structure of the small molecule fragment binder found by STD spectroscopy was used as a constraint for the next search procedure. We searched the rapidly growing ZINC database^15^, which contains the structures of over 100 million compounds, for those with at least 70% similarity to the fragment molecules identified by STD spectroscopy. The two fragment molecules ranked highest by the fragment based screening – indole and 2H-chromene-3-carbothioamide – were used for this step of virtual filtration. The database search yielded approximately 3000 compounds per scaffold. We then used the docking software AutoDock^16^ to dock these compounds into the active site of T7 primase using its available crystal structure (pdb code 1nui^17^). The compounds were then ranked based on their ΔG predicted binding energy values. The highest-ranking compounds that contained indole or 2H-chromene-3-carbothioamide were obtained from the ZINC database for functional and structural assays with the target T7 primase (Fig. S1).

### Small molecules that inhibit the concerted activity of primase and DNA polymerase

**Figure 4:**
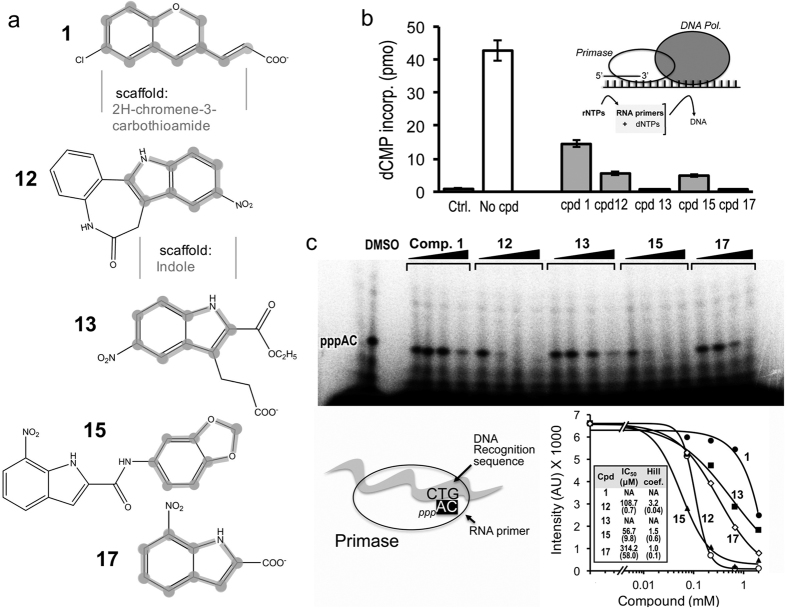
Small molecule inhibitors that contain fragments obtained by STD spectroscopy. **(a)** Chemical structures of five small molecules obtained by virtual filtration using the ZINC database^15^ and high-throughput docking using AutoDock^16^ (list of 16 compounds presented in Fig. S1). The two subsets are based on the scaffolds obtained by STD spectroscopy: 2H-chromene-3-carbothioamide and indole (emphasized in thick grey). (**b**) Inhibitory effect of small molecules on bacteriophage T7 primase. Primase-dependent DNA synthesis. The reaction contained 0.3 mM dATP, dGTP, dCTP and [α–^32^P] dTTP (0.1 μCi), 100 μM ATP and CTP, 10 nM gp5/trx, 200 nM monomeric concentration of gp4A, 10 nM M13 ssDNA and 350 μM of each of the compounds. The reaction mixture was incubated for 30 min at 37 °C and spotted on DE81 filter paper. The amount of radioactivity remaining on the filter paper was measured (inset presents experimental setup). The amounts of RNA–primed DNA syntheses were determined by measuring the incorporation of dTMP (see Methods). The error bars were derived from three independent experiments. (**c**) Template-directed pppAC ribonucleotide synthesis catalyzed by T7 DNA primase. Reaction conditions involve incubating the primase domain with an oligonucleotide containing a primase recognition sequence. In this assay (bottom left), the DNA template containing the primase recognition site 5′-GTCA_10_-3′ enables the synthesis of only diribonucleotides pppAC. The reaction also contained [α-^32^P] CTP, ATP, and increasing amounts of the tested compounds (1.1, 3.3 and 10 μM). After incubation, the radioactive products were analyzed by electrophoresis through a 25% polyacrylamide gel containing 3 M urea and visualized using autoradiography (Fig. S2b presents results for all compounds). Bottom right: Quantification of gel bands representing the reaction products (5′-pppAC-3′).

To evaluate the ability of the small molecule candidates to inhibit the primase-dependent replication of the lagging strand of bacteriophage DNA, we ran an overall assay that involved the concerted activity of DNA polymerase (gene product 5 and *E. coli* thioredoxin, gp5/trx) and the helicase-primase (gene product 4, gp4, full-length). In bacteriophage T7, lagging strand DNA synthesis involves interactions between gp5/trx and gp4. Full-length gp4 is required for the synthesis of oligoribonucleotides to initiate the synthesis of Okazaki fragments^5^. To examine the effect of 350 μM of each small molecule on the synthesis of primers and their transfer to gp5/trx, we used M13 ssDNA for the synthesis of oligoribonucleotides by the primase and their extension by gp5/trx (Fig. 4b, inset). To initiate DNA synthesis, the primase must first synthesize tetraribonucleotides on the DNA and then transfer them to gp5/trx. In addition to the four dNTPs, ATP and CTP were also provided, and the primers that were synthesized included pppACCC, pppACAC, and pppACCA. Inhibition of the primase activity of gp4 halts RNA primer formation process, thereby preventing subsequent DNA polymerase activity. Figure 4 shows that primase dependent DNA synthesis decreases by up to four-fold with the addition of five small molecules, including (2E)-3-(6-chloro-2H-chromen-3-yl)acrylic acid (compound 1), 9-Nitro-7,12-dihydroindolo-[3,2-d][1]benzazepin-6(5)-one (compound 12), 3-[2-(ethoxycarbonyl)-5-nitro-1H-indol-3-yl]propanoic acid (compound 13), N-(1,3-benzodioxol-5-yl)-7-nitro-1H-indole-2-carboxamide (compound 15), and 7-nitro-1H-indole-2-carboxylic acid (compound 17), whose chemical structures are presented in Fig. 4a.

### Small molecules that inhibit primase activity

To catalyze the synthesis of short RNA primers, T7 primase requires DNA with a primase recognition site, ATP and CTP, and a buffer containing divalent metal ions. The effect of each of the five small molecules found using FBVS approach as described above on RNA primer synthesis by the T7 primase was examined. The primase domain catalyzed the synthesis of the diribonucleotide pppAC on a DNA template containing the 5′-GTC-3′ primase recognition site^5^. We examined diribonucleotide synthesis in the presence of each of the five small molecules found to inhibit the concerted activity of primase and polymerase (Fig. 4b). The reaction conditions involved incubating the T7 primase with an oligonucleotide containing a primase recognition sequence, [α-^32^P]-CTP and ATP, and adding each small molecule in steadily increasing amounts. The radioactively labeled oligoribonucleotides were separated on a denaturing polyacrylamide gel, and radioactivity was measured on an autoradiogram. The results clearly show that inhibition of the specific activity of the primase increased with increasing amounts of the small molecules (Fig. 4c). Curve fitting was performed using nonlinear four parameter logistics to explore features of small molecule binding to the enzyme. Values for IC_50_ and Hill coefficient were extracted (Fig. 4c, right bottom). Data for compounds 1 and 13 were not sufficient for the analysis therefore IC_50_ values and Hill coefficients were excluded. Overall, IC_50_ for those molecules were in the sub-millimolar range. Hill coefficient for compound 12 present highest value presumably due to stronger binding of the molecule to the enzyme.

### Structural analysis of inhibitor-primase interactions

**Figure 5:**
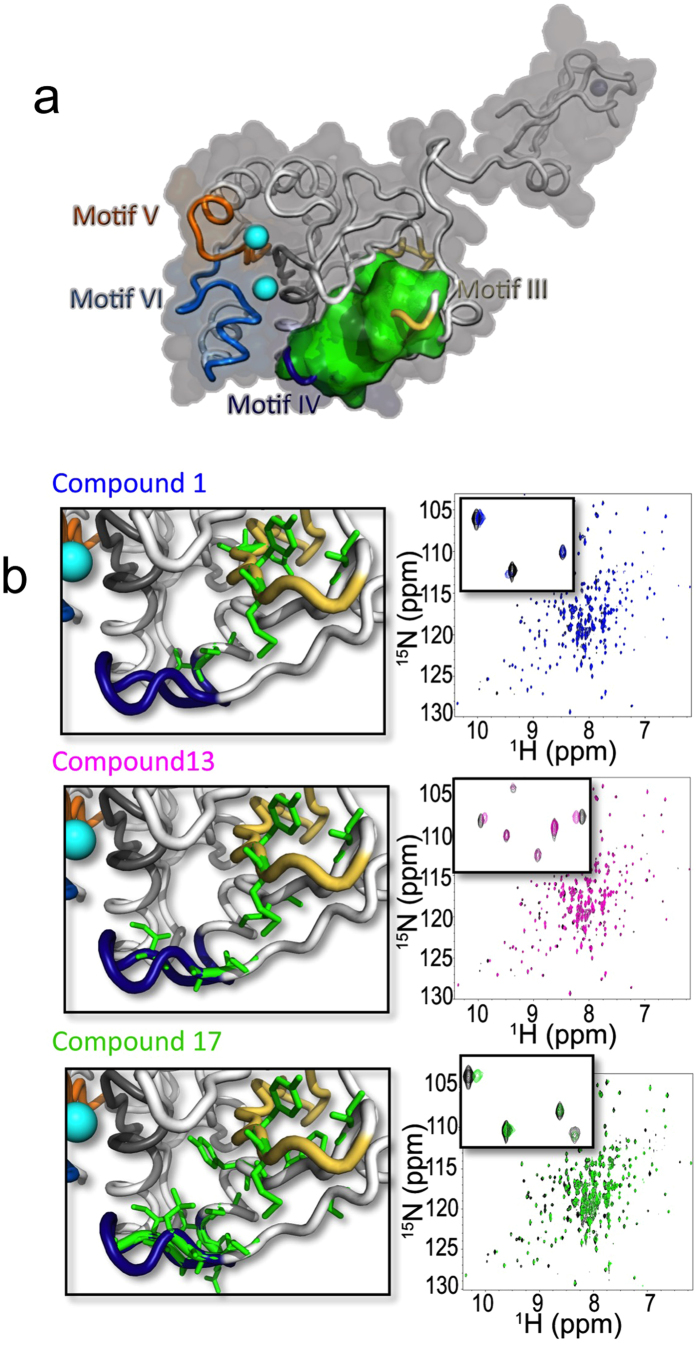
Binding-site of small-molecule inhibitors on T7 DNA primase. **(a)** Amino acid sequence chemical shift assignments indicate that small molecule binding occurs in the proximity of the main cleft of T7 DNA primase (binding site indicated in green). (**b**) Right: Two-dimensional ^1^H-^15^N HSQC spectrum of T7 DNA primase alone (black spots) and in the presence of each small molecule inhibitor (blue, compound 1; purple, compound 13; green, compound 17). The HSQC spectrum of primase changes in the wake of small molecule binding. Similar peaks change upon titration of DNA (GGTCA) or ATP and CTP in the presence of magnesium added in a ratio molar concentration. Left: Amino acid residues that mediate the binding of each small molecule inhibitor are indicated in green.

To characterize the binding of the compounds identified using FBVS, we measured the [^15^N, ^1^H]-TROSY-HSQC spectra of ^15^N- and D-labeled T7 primase in the presence of the selected lead compounds (Fig. 5). After the addition of compounds 1, 13, and 17 to T7 primase, we observed significant chemical shift perturbations compared to those obtained for the free T7 primase. Relative to the initial scaffold alone, those shifts were more pronounced, which indicates stronger binding interactions. In addition, chemical shift perturbations upon the addition of DNA and ATP/CTP to the T7 primase domain confirmed that all three compounds bind to the active site. Moreover, the presence of several cross peaks among the three compounds indicates that they exploit a similar binding mechanism. Compounds 12 and 15, whose polar dissolution values were the lowest from among the five inhibitors, were not able to achieve the concentrations needed for the protein-NMR experiments.

To unravel the binding site of T7 primase inhibitors, we assigned the resonances to the T7 primase backbone (*see* Methods). With the exception of the zinc-binding domain, 70% of the chemical shifts of the T7 primase have been assigned to their corresponding residues (unpublished data). Assignment of the NMR peaks to the protein residues enabled us to identify the amino acid residues situated in the proximity of the active site that mediate small molecule inhibitor binding (Fig. 5a,b). Protein stability was severely impaired if the six amino acid residues at the binding site – Ala(80), Ser(87), Glu(89), Val(101), Met(105), Tyr(106) – are substituted altogether with Ala (except Ala(80), which was substituted with Gly).

**Figure 6:**
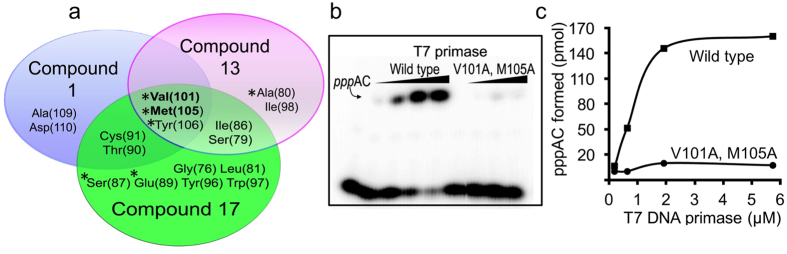
Essentiality of amino acids that mediate the binding of small molecule inhibitors. (**a)** Amino acid residues that mediate small molecule inhibitors at the active site of T7 DNA primase. The three T7 primase inhibitors share the same binding site and are mediated via similar binding mechanisms. Solvent accessible amino acid residues were calculated using Naccess (http://www.bioinf.manchester.ac.uk/naccess/) and are marked in asterisk. (**b**) Substitutions of the central amino acids that were shown to mediate the binding of all the tested inhibitors and are solvent accessible (i.e., Val101 and Met105 were replaced with Ala) disrupted protein activity. The faint signal of pppAC remained for the reaction of the double mutant T7 DNA primase may be due to erroneous loading of the sample into the gel. Reaction conditions were as in Fig. 4 (except that the protein concentrations were 0.2, 0.6, 1.9, 5.8 *μ*M, respectively). (**c**) Quantification of di-ribonucleotide synthesis by T7 DNA primase. The bands in the gels presented in b were analyzed using autoradiography.

Figure 6a shows the amino acid residues that mediate the binding of each of the three small molecule inhibitors [(2E)-3-(6-chloro-2H-chromen-3-yl)acrylic acid (compound 1); 3-[2-(ethoxycarbonyl)-5-nitro-1H-indol-3-yl]propanoic acid (compound 13); 7-nitro-1H-indole-2-carboxylic acid (compound 17)]. Indeed, for each of the three compounds, the mechanism of binding to the active site cleft is similar but not identical, and the amino acids involved overlap slightly, such that Val(101), Met(105), and Tyr(106) mediate the binding of all three compounds (Fig. 6a). All three of the small molecules bind to the primase active site and are expected to interfere with the binding to the substrate (ribonucleotide tri-phosphates) or to the DNA template. Indeed, substituting two of the amino acid residues that mediate the binding of all three compounds [Val(101) and Met(105)] with Ala inactivated the primase (Fig. 6), an outcome that is indicative of the central roles these two amino acid residues play in primer synthesis. More importantly, this specific binding location represents a potential route to prevent bacteria from evolving into a resistant strain: any adaptive mutation driven by inhibitor binding would completely disrupt primase activity, killing the bacteria as a result.

## Discussion

Functional assays for primase generate weak readout signals, and therefore, are not easily adapted to high throughput screening. The T7 primase is an ideal model to study bacterial primases because (1) it shares high structural similarity with bacterial primases, (2) it is highly expressed, (3) it has been extensively studied, and (4) its crystal structure is known. We assigned NMR HSQC perturbations to the amino acid sequences of the primase, which enabled us to identify the amino acid residues that mediate small molecule inhibitor binding. This knowledge will be invaluable for further optimization and structure activity relationship studies, which have the potential to lead to the development of new antibacterial drugs.

In this study, we introduced FBVS, a novel analytical technique that combines a spectroscopic assay (STD spectroscopy) with an *in-silico* approach. Its computational component comprised the virtual filtration of a small molecule database followed by the docking of the potential small molecule inhibitors to the crystal structure of T7 primase. Not only did this approach allow us to find several lead compounds for T7 primase inhibition, it also showed a high hit rate: of the 16 compounds ordered for testing, about half showed primase inhibition activity. This approach, which is not only fast but also cost effective, is a promising method for identifying the inhibitors of protein targets that are not amenable to high throughput screening.

The fragment based screening approach proposed here enabled us to rationally design drug like inhibitors (that contain small fragments) to the primase. However, in contrast to conventional fragment-based screening, which requires a subsequent medicinal chemistry step to grow the hit molecules, FBVS eliminates the need for that step early on and increases the chances of successfully identifying larger molecules containing the fragment hits.

Insofar as the inhibition of DNA primase will stop bacterial DNA replication and prevent infection, the primase domain of phage T7 gene 4 product represents an important drug target. We therefore expect that a T7 DNA primase inhibitor will be at the forefront of a new class of anti-bacterial agents.

## Methods

### Protein expression and purification

Chemicals were from Sigma. ATP and CTP were from Roche Molecular Biochemicals. dNTPs were from USB Corp. Pre-made gels (10-20% linear gradients) were from BioRad (Hercules, CA). T7 gp5, gp4, *E. coli* trx were overproduced and purified as described^18^,^19^. M13 ssDNA was prepared as described previously^20^. [γ–^32^P] dATP (800 Ci/mmol), [α–^32^P] CTP, and dTTP (800 Ci/mmol) were from Perkin Elmer.

### Protein expression and purification

Proteins and Reagents. All chemical reagents were of molecular biology grade (Sigma); ATP and CTP (Roche Molecular Biochemicals). dNTPs and ddNTPs were purchased from USB Corp. Premade gels (10–20% linear gradients) used for SDS–PAGE and Precision Plus Protein prestained standards were purchased from BioRad (Hercules, CA). T7 primase domain (residue: 1–271) was over-produced and purified using metal free buffers as previously described^17^,^21^. T7 gp5 and E. Coli trx were overproduced and purified as described^18^. Gp4 was overproduced and purified as described^19^. M13 ssDNA was prepared as described previously^20^. [α–^32^P]–CTP (800 Ci/mmol) was purchased from Perkin Elmer.

### Virtual screening

The hits determined by NMR were used to identify compounds with at least 70% similarity from the ZINC database^15^. Virtual screening was performed to identify molecules that could bind in the active site of T7 DNA primase and inhibit DNA replication. Docking of all compounds downloaded from the ZINC database was performed using Autodock4.2.3^16^. PDB files of the receptor (T7 primase) and the ligands (compounds) were prepared prior to docking as described in the tutorial “Using AutoDock for Virtual Screening” (http://autodock.scripps.edu/faqs-help/tutorial/using-autodock-for-virtual-screening/UsingAutoDockforVirtualScreening_v7.pdf). The search grid was centered in the active site of T7 primase with a grid spacing of 0.375 Å and 110 × 108 × 126 points. The default parameters were used except for the following modified parameters: ga_num_evals = 1750000, ga_pop_size = 150, and ga_run = 100.

### Primase dependent DNA synthesis

RNA primers made by gp4A were extended by gp5/trx. The reaction mixture contained 10 nM M13 ssDNA, 0.3 mM dNTPs, 0.1 μCi [α–^32^P] dCTP, 20 nM gp5/trx, 200 nM monomeric gp4A, and 350 μM of each compound. The reaction was incubated for 45 min at 37 °C. Reaction was terminated and amount of DNA synthesis was determined as described in DNA polymerase assay.

### DNA polymerase assay

DNA polymerase activity was measured in a reaction containing 5 nM gp5/trx, 20 nM M13 ssDNA annealed to a 24mer primer, 40 mM Tris–HCl (pH 7.5), 10 mM MgCl_2_, 10 mM DTT, 50 mM potassium glutamate, 0.25 mM dTTP, dGTP, dCTP, [α–^32^P] dATP (5 cpm/pmol) and 500 μM of each compound. The reaction was incubated at 37 °C for 20 min and terminated by the addition of EDTA to a final concentration of 40 mM. Aliquots of the reaction were spotted on DE–81 filters (Whatman), washed three times with excess of 0.3 M ammonium formate (pH 8.0), and the radioactivity retained on the filters measured.

### Oligoribonucleotide Synthesis Assay

Oligoribonucleotides were synthesized by DNA primase, product was measured as described^22^,^23^ in reactions containing various concentrations (1.1, 3.3, and 10 μM) of gene 4 primase domain. Standard 10 μL reaction contained 5 μM of DNA template (5′-GGGTCA_10_-3′), 200 μM ATP, 200 μM [α-^32^P]-CTP, and primase domain in a buffer containing 40 mM Tris-HCl (pH 7.5), 10 mM MnCl_2_, 10 mM DTT, and 50 mM potassium glutamate. After incubation at room temperature for 20 minutes, the reaction was terminated by adding an equal volume of sequencing buffer containing 98% formamide, 0.1% bromophenolblue, and 20 mM EDTA. The samples were loaded onto 25% polyacrylamide sequencing gel containing 3 M urea and visualized using autoradiography.

### Sample preparation

For the expression of unlabeled proteins, the primase domain of the bacteriophage T7 gene 4 product (1–271) was expressed in *E. coli* Bl21(DE3) containing pETg4P as reported previously^21^. For expression of isotopically enriched proteins, a starter culture was used to inoculate 2 liters of M9 medium containing 50 μg/mL kanamycin, 1 g ^15^N-NH_4_Cl, and 2 g ^13^C-glucose or 2 g ^2^H,^13^C-glucose. The M9 was made up in D_2_O for expression of perdeuterated proteins. When the culture optical density (600 nm) reached approximately 0.8, the culture was induced for protein overexpression with 0.3 mM isopropyl-β-D-thiogalactopyranoside (IPTG) for 16 to 24 h at 16 °C. Cell pellet was resuspended in buffer A (50 mM Tris-HCl pH = 7.5, 1 mM EDTA, 1 mM DTT), 100 mM NaCl, 0.25 mg/ml lysozyme and 1 mM PMSF and incubated in ice for 1 h. After four cycles of freeze-thaw streptomycin sulfate was added to a concentration of 1%. After centrifugation at 15,000× g for 30 min supernatant was diluted with Buffer A (no salt) fivefold and loaded onto DEAE sepharose (packed in AP-5, Waters, NJ) and washed with 200 mL of 50 mM Tris-HCl. Primase domain was eluted using linear gradient of Buffer A from no salt to 1 M NaCl. Primase fractions were combined and ammonium sulfate was added (0.361 g/ml). The precipitant was collected by centrifugation (15,000× g for 30 min), dissolved in 10 mL buffer A, loaded onto a sephacryl S-200HR column and eluted using Buffer A. The fractions containing the primase domain were combined and applied to a 5-ml HiTrap Blue affinity column. The column was washed with excess amount of buffer A and the primase was eluted using a linear gradient of NaCl (0–1 M) in Buffer A. The pure protein was dialyzed against buffer B (50 mM KH_2_PO_4_/K_2_HPO_4_ pH = 7, 1 mM DTT) and concentrated to 0.4 mM.

### NMR experiments –Fragment Based Screening

T7 primase was screened against the Maybridge Ro3 Diversity Fragment Library containing 1000 compounds using STD spectroscopy. Samples for 1D ligand-observed NMR studies contained 5 μM T7 primase in 50 mM phosphate buffer in D_2_O and 10 compounds, resulting in a total of 100 samples. NMR spectra were recorded on a Bruker Avance 500 MHz equipped with a TXO cryoprobe with Z gradient and a NMR-CASE sample changer at 298 K. Ligand binding was probed using a saturation transfer difference pulse program^24^,^25^,^26^. Saturation was achieved using on-resonance irradiation at 0 ppm with a train of Wurst pulses for a total saturation time of 1.5 s. Off resonance irradiation was centered at 40 ppm. Spectra were acquired with a sweep width of 8012.8 Hz, 8192 data points and 224 scans. Active compounds were identified by comparing the STD spectra with the corresponding reference spectra using the MestreNova software. Any compound with reduced peak intensities was considered a hit and ranked according to the percentage of intensity decrease and the number of affected peaks per compound.

[^1^H,^15^N]-TROSY HSQC titration spectra of ^15^N,D T7 DNA primase with selected fragment molecules and compounds 1, 12, 13, 15, 17, ATP, and DNA were recorded at 25 °C on a Bruker DMX 800 MHz spectrometer equipped with TXI cryoprobes with Z gradient. Data were processed and analyzed using NMRPipe^27^ and NMRView^28^.

### NMR experiments – Backbone resonance assignments

Spectra were acquired at 298.15 K with 700 μM protein samples in 25 mM KH_2_PO_4_/K_2_HPO_4_, pH = 7.2, 150 mM NaCl, 1 mM DTT. Traditional TROSY-based backbone triple resonance experiments (HNCA/HNCOCA, HNCO/HNCACO, HNCACB) were conducted on a ^15^N,^13^C-perdeuterated sample to assign the backbone chemical shifts. The spectra were recorded on a Varian 600 MHz spectrometer equipped with cryogenically cooled probe. Non Uniform Sampling was used in all the triple resonance experiments where 12% of the indirect dimension grid was sampled and the spectrum was reconstructed using hmsIST^29^ and NMRPipe^30^. The resulting spectra were visualized and analyzed using CARA^31^.

## Additional Information

**How to cite this article:** Ilic, S. *et al.* Identification of DNA primase inhibitors via a combined fragment-based and virtual screening. *Sci. Rep.* **6**, 36322; doi: 10.1038/srep36322 (2016).

**Publisher’s note:** Springer Nature remains neutral with regard to jurisdictional claims in published maps and institutional affiliations.

## Supplementary Material

Supplementary Information

## Figures and Tables

**Figure 2:**
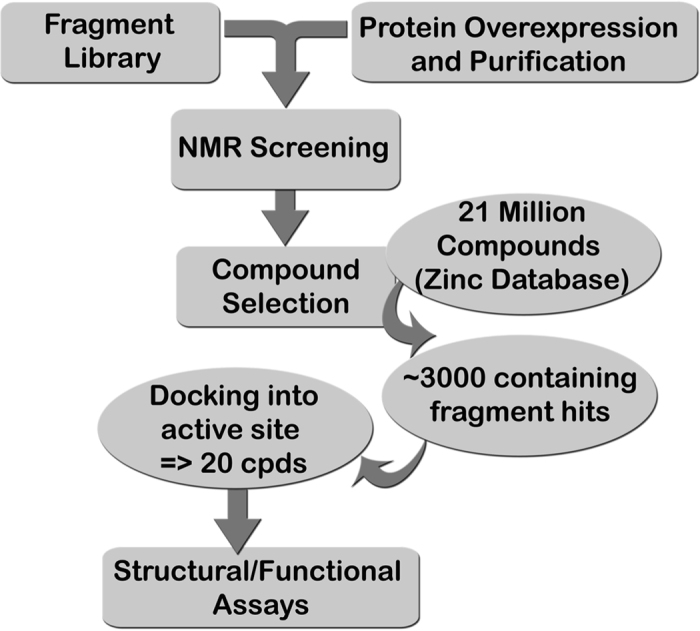
Fragment based virtual screening. An approach combining NMR-fragment based screening with virtual screening to select inhibitors against phage T7 DNA primase. Using 1D NMR (STD) and a fragment library, we identified scaffolds that bind T7 DNA primase. These scaffolds were used as a filter to select larger compounds with drug-like properties from a database of 21 million compounds (ZINC). Thousands of compounds for each scaffold were docked to the active site of T7 DNA primase (pdb code: 1nui) and hits were ranked based on the binding energy. Several candidate compounds were selected and tested for their ability to inhibit T7 DNA replication.
