# Supplementary material for: Identification of DNA primase inhibitors via a combined fragment-based and virtual screening
Source: Sci Rep. 2016 Nov 2;6:36322. doi: 10.1038/srep36322 (PMC5090872; doi:10.1038/srep36322)
Supplement: Supplementary Information [file srep36322-s1.pdf]

Supplementary Information

Identification of DNA primase inhibitors via a combined fragment-based and virtual screening

Stefan Ilic[1], Sabine R. Akabayov[1,2], Haribabu Arthanari[2], Gerhard Wagner[2], Charles C. Richardson[2] & Barak Akabayov[1*]

[1] Department of Chemistry, Ben-Gurion University of the Negev, Beer-Sheva, 8410501 Israel

[2] Department of Biological Chemistry and Molecular Pharmacology, Harvard Medical School, 240 Longwood Ave., Boston, Massachusetts 02115, USA.

[*]email: akabayov@bgu.ac.il

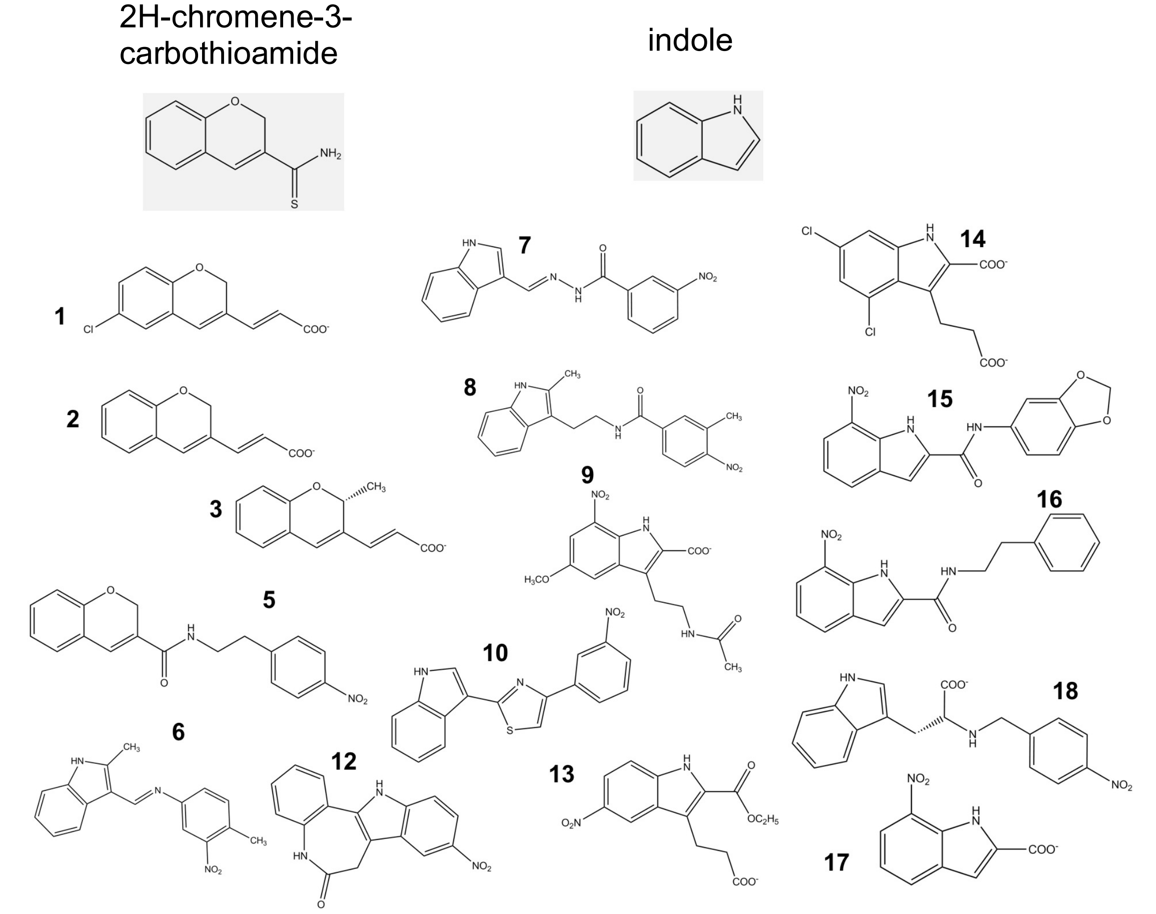

Figure S1 Chemical structures of 16 small molecules obtained by virtual filtration using the ZINC database[17] and high-throughput docking using AutoDock[18]. The two subsets are based on the scaffolds obtained by STD spectroscopy: 2H-chromene-3-carbothioamide and indole (indicated in grey).

a

Protein	DNA template	Substrate
Helicase/Primase Polymerase/trx (Replisome)	Single stranded DNA (M13)	ATP/CTP dNTPs
Primase fragment	GGGTCA10	ATP/CTP

b

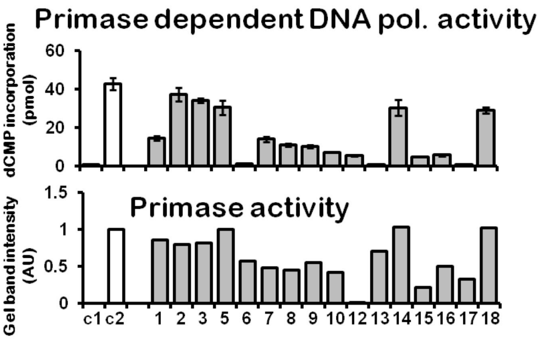

Figure S2 Inhibitory effect of small molecules on T7 DNA primase activity. (**a**) Effect of small molecule inhibitors on primase-dependent DNA synthesis mediated by gp5/trx and gene 4 helicase (gp4A). The standard reaction contained the ssM13 DNA template (10 nM), 0.3 mM dATP, dGTP, dCTP and [α-^{32}P] dTTP (0.1 mCi), 10 nM gp5/trx, 200 nM monomeric concentrations of gp4B and 250 μM of small molecule inhibitors. After incubation for 30 min at 37 °C, the level of RNA synthesis was determined based on the amount of [α-^{32}P] dTTP incorporated into DNA. (**b**) Effect of small molecule inhibitors on oligonucleotide synthesis by DNA primase. The standard reaction contained the oligonucleotide 5'-GGGTCA$_{10}$-'3 containing the primase recognition sequence, 200 mM [α-^{32}P]-CTP and ATP, and 250 μM small molecule inhibitors in a buffer containing 40 mM Tris-HCl (pH 7.5), 10 mM MnCl2, 10 mM DTT and 50 mM potassium glutamate. The quenched samples were loaded onto 25% polyacrylamide sequencing gel containing 3M urea and visualized using autoradiography.
